# Studying Dynamical Characteristics of Oxygen Saturation Variability Signals Using Haar Wavelet

**DOI:** 10.3390/healthcare11162280

**Published:** 2023-08-13

**Authors:** Madini O. Alassafi, Ishtiaq Rasool Khan, Rayed AlGhamdi, Wajid Aziz, Abdulrahman A. Alshdadi, Mohamed M. Dessouky, Adel Bahaddad, Ali Altalbe, Nabeel Albishry

**Affiliations:** 1 Faculty of Computing and Information Technology, King Abdulaziz University, Jeddah 21589, Saudi Arabia; malasafi@kau.edu.sa (M.O.A.); dbabahaddad10@kau.edu.sa (A.B.); aaltalbi@kau.edu.sa (A.A.); nalbishry@kau.edu.sa (N.A.); 2 College of Computer Science and Engineering, University of Jeddah, Jeddah 21725, Saudi Arabia; irkhan@uj.edu.sa (I.R.K.); alshdadi@uj.edu.sa (A.A.A.); mmdessouky@uj.edu.sa (M.M.D.); 3 Department of Computer Science and Information Technology, King Abdullah Campus, University of Azad Jammu and Kashmir Muzaffarabad (AK), Azad Jammu and Kashmir 13100, Pakistan; kh.wajid@ajku.edu.pk; 4 Department of Computer Science & Engineering, Faculty of Electronic Engineering, Menoufia University, Menouf 12548, Egypt

**Keywords:** biomedical signal processing, COVID-19, Haar wavelet, oxygen saturation variability, physiological systems

## Abstract

An aim of the analysis of biomedical signals such as heart rate variability signals, brain signals, oxygen saturation variability (OSV) signals, etc., is for the design and development of tools to extract information about the underlying complexity of physiological systems, to detect physiological states, monitor health conditions over time, or predict pathological conditions. Entropy-based complexity measures are commonly used to quantify the complexity of biomedical signals; however novel complexity measures need to be explored in the context of biomedical signal classification. In this work, we present a novel technique that used Haar wavelets to analyze the complexity of OSV signals of subjects during COVID-19 infection and after recovery. The data used to evaluate the performance of the proposed algorithms comprised recordings of OSV signals from 44 COVID-19 patients during illness and after recovery. The performance of the proposed technique was compared with four, scale-based entropy measures: multiscale entropy (MSE); multiscale permutation entropy (MPE); multiscale fuzzy entropy (MFE); multiscale amplitude-aware permutation entropy (MAMPE). Preliminary results of the pilot study revealed that the proposed algorithm outperformed MSE, MPE, MFE, and MMAPE in terms of better accuracy and time efficiency for separating during and after recovery the OSV signals of COVID-19 subjects. Further studies are needed to evaluate the potential of the proposed algorithm for large datasets and in the context of other biomedical signal classifications.

## 1. Introduction

The human body is comprised of several complex systems composed of the functions of several organs that work coherently in a healthy human. Different functions or malfunctions of organs caused by disease affect several vital signs, which are very helpful in diagnosing the disease [1,2]. Complexity analysis of medical signals involves the quantitative assessment and characterization of the intricate patterns and dynamics present in physiological signals [3,4]. It aims to unveil hidden information and provide insights into underlying physiological processes and their changes in various health conditions. Complexity analysis offers a framework to investigate medical signal complexity, irregularity, and non-linear dynamics by employing mathematical and computational techniques [5,6].

Complexity analysis of medical signals has found applications in various fields such as cardiology, neurology, respiratory medicine, and sleep research. It has contributed to the development of advanced signal processing methods, including nonlinear dynamics, recurrence analysis, fractal analysis, and complexity-based machine learning algorithms. By uncovering hidden patterns and changes in medical signals, complexity analysis holds the potential to enhance disease diagnosis, monitor disease progression, predict patient outcomes, and guide personalized treatment strategies [7,8].

Overall, complexity analysis of medical signals plays a crucial role in deepening our understanding of physiological systems, unraveling the complexity of diseases, and improving healthcare practices by providing valuable quantitative insights into the intricate dynamics of physiological processes [9].

Entropy-based measures such as approximate entropy [10], sample entropy [11], permutation entropy [12], and fuzzy entropy [13] have been used in numerous studies for quantifying the complexity of the underlying controlling mechanism of physiological and non-physiological systems. The approximate entropy, sample entropy, and fuzzy measures quantify signal complexity by examining the predictability and pattern regularity within the data, whilst permutation entropy quantifies the complexity of the signals based on ordinal patterns. The signals from complex physiological systems are non-linear, non-stationarity, and time irreversible while exhibiting at multiscale. These traditional single-scale entropy measures do not take into account multiple temporal scales and thus yield contradictory results about the dynamical characteristics of the real-world signals of physiological and pathophysiological systems. Multiscale entropy (MSE) [14] analysis is another important technique that assesses complexity across different temporal scales, enabling the detection of scale-dependent patterns and dynamics [15].

Respiratory and cardiovascular systems maintain adequate levels of oxygen for the healthy working of the body, and measurement of oxygen saturation (SpO2) is often used as an indication of certain diseases. Analysis of oxygen saturation variability (OSV) has been used to test the integrity of the cardio–respiratory control system (CRCS). The studies [16,17,18,19] revealed that information exchange between respiratory variables and OSV signals can provide valuable insights into the dynamical complexity of CRCS with changes occurring due to aging and pathological conditions. The study [16] reported MSE values were smaller for elderly subjects at all temporal scales, revealing not only loss of complexity but also the de-coupling of the controlling mechanism of CRCS. The entropy analysis of OSV signals was used by researchers to study the effect of normobaric hypoxia and earlier detection of exacerbations in chronic obstructive pulmonary disease in individuals [17,18,19]. 

The entropy-based complexity measures along with their multiscale version are commonly used metrics for analyzing the dynamics of physiological systems and can have the potential to assess the complexity of OSV signals of COVID-19 subjects. Multiscale entropy measures differ in computational details and are more robust and accurate in capturing the dynamical characteristics of physiological signals. However, the multi-scale analysis adds to the computational burden and slows the process of analyzing the complexity of OSV signals [20]. The aim of this study was to develop a sophisticated, reliable, and efficient entropy measure for quantifying the complexity of OSV signals acquired from COVID-19 subjects. This research work proposed a novel technique using Haar wavelets to analyze the complexity of OSV signals of COVID-19 patients during and after recovery. Haar wavelet is a sequence of rescaled “square-shaped” functions forming a wavelet family or basis. It is the simplest, and least regular type of Daubechies wavelets, reproducing constant functions only. The Haar scaling function is represented with a simple formula, whilst scaling functions for other Daubechies wavelets are developed from the dilation equation using the cascade algorithm. Haar wavelets are discrete and can be used for analyzing signals with transitions [21]. In this study Haar Transform was used to convert OSV time series into two trend and fluctuation sub-signals. We iteratively applied this function five times to obtain the sub-signals up to stage 5. The fluctuation signals were discarded and we considered only fluctuation signals for analyzing OSV time series because the variations in the trend signal represent well the complexity of the signal. All five sub-signals were normalized to zero-mean for eliminating the bias and the method of counting zero-crossings was used for measuring the variability in each of the zero-mean trend sub-signals. The mean value was taken as the overall score of the structural fidelity of the signal. The accuracy and time efficiency of the proposed technique was compared with well-known complexity measures MSE [14], MPE [22], MFE [23], and MAAPE [24] during COVID-19 and after two months of recovery.

## 2. Literature Review

Complexity analysis of physiological signals in the understanding of the functioning of various human body systems has been a popular research area. PhysioBank, PhysioToolkit, and PhysioNet are interconnected resources that provide open-access physiological data, software tools, and educational materials in the field of biomedical engineering [25]. These are very valuable resources of complex physiologic signals which can be analyzed to gain insights into the function of human body systems.

Several works have focused on the analysis of physiological signals in understanding their complex dynamics and their relevance in characterizing physiological processes by considering chaos as an underlying model [26,27,28,29]. Peng et al. [26] examined the mosaic organization of DNA nucleotides, revealing complex patterns within DNA sequences and hidden relationships and complex characteristics in genetic information. Ivanov et al. [27] explored the scaling behavior of heartbeat intervals using wavelet-based time-series analysis, revealing the multifractal nature of heart rate variability. Ivanov et al. [28] investigated the scaling and universality in heart rate variability distributions, uncovering the presence of scaling behavior and universal patterns. Goldberger [29] discussed the application of non-linear dynamics, chaos theory, fractals, and complexity analysis in clinical settings, emphasizing their potential impact on clinical understanding and decision-making.

Entropy is a measure used to quantify the randomness or uncertainty in a time series [10,11]. It provides information about the distribution and predictability of data patterns and gives a measure of the complexity of the data [10,11,12]. In 1991 Pincus [10] proposed approximate entropy to determine the complexity of deterministic chaos and stochastic processes comprising at least 1000 data points. The capability of approximate entropy to discern the complexity of a small number of data points has been explored in numerous fields, especially in medical sciences. Sample entropy [11], a modified version of approximate entropy is used to quantify the complexity and irregularity of a time series by assessing the likelihood of occurring similar patterns, which holds promise for analyzing biomedical signals and physiological data. Fuzzy entropy is a measure used to assess the complexity and irregularity of a time series based on the fuzzy membership function approach [13]. It examines the likelihood that two vectors, which exhibit similarity over a certain number of points (m), will continue to remain similar for the subsequent (m + 1) points.

Bandt and Pompe proposed permutation entropy [12], to analyze the patterns obtained by permuting the order of values and to provide insights into the irregularity and unpredictability of the data. Permutation entropy does not consider the average amplitude values and equal amplitude values in the symbolized time series. Azami and Escudero [30] proposed amplitude-aware permutation entropy to address the issue of amplitude information in the formulation of permutation entropy. Threshold-dependent symbolic entropy was proposed by investigators to estimate the complexity of stride interval time series [31]. Porta et al. [32] furthered understanding by employing entropy and pattern classification techniques to characterize complexity in short heart period variability series, aiding in the classification of cardiac states. Lake and Moorman [33] addressed the challenge of accurately estimating entropy in a very short physiological time series, with a specific focus on the detection of atrial fibrillation in implanted ventricular devices.

The traditional single-scale entropy measures fail to account for multiscale variability inherent in the physiological signals and can yield contradictory results [14,22]. Multiscale entropy (MSE) extends the concept of entropy by considering different time scales. By analyzing the entropy values across different time scales, multiscale entropy provides a more comprehensive understanding of the dynamics and complexity of the time series at various levels of detail [14]. The different versions of scale-based entropy metrics have demonstrated successful applications across various domains. However, it faces a challenge when estimating the statistical reliability of sample entropy for coarse-grained series as the time scale factor increases. Investigators proposed other scale-based entropy measures such as MPE [22], MFE [23], and MAAPE [21] to account for shortcomings of MSE and applied them in numerous fields.

In several recent studies, researchers quantitatively described oxygen saturation dynamics and patterns in COVID-19 patients [34,35,36]. In [34] researchers performed retrospective observational analysis of the clinical information and high-resolution oxygen saturation recordings of 367 hospitalized critical and non-critical COVID-19 patients. The findings revealed that the oximetry-derived digital biomarkers were informative for assessing the disease severity and response to respiratory support of hospitalized COVID-19 patients. Zhang et al. [35] proposed a method to assesses the subject’s degree of illness considering the supply chain and Industry 5.0 requirement and calculated the apnea-hypopnea index (AHI) and the subject’s disease level. They used oxygen saturation signals to extract features based on the time domain, central tendency measure, approximate entropy, and Lempel–Ziv complexity. Using a random forest classifier, 92% accuracy was obtained in assessing the prevalence of obstructive sleep apnea–hypopnea syndrome. In [36], authors used a mathematical approach to describe the exercise ventilatory responses in patients with dyspnoea and breathing pattern disorder following COVID-19. Aliani et al. [37] conducted a study to investigate heart rate variability (HRV) of healthy subjects and COVID-19 patients and reported significant differences between the groups in several HRV parameters.

## 3. Methodology

### 3.1. Dataset

The data used in the study comprises 44 recordings (26 male and 18 female) of oxygen saturation SpO2 acquired from confirmed COVID-19-infected patients and 44 recordings two months after recovery. Eleven subjects (25%) were diabetic and 18 (40.91%) were hypertensive. Five subjects (11.36%) were non-smokers and 37 (86.36%) were vaccinated. About 93 (40.91%) subjects suffered from COVID-19 infection once and about 7.0% twice. A Beurer PO-80 oximeter, which is a noninvasive device was used for recording SpO2 [38]. The integrated recording function of Beurer PO-80 provides continuous recording of up to 24 h [38]. The Beurer View and Evaluate SpO2 Assistant free software enables the device to synchronize with personal computers via USB. The necessary protocols were followed before acquiring data from patients during COVID-19 infections. The patients were briefed about the purpose of the study, and informed consent was taken for data collection. The University of Azad Jammu & Kashmir Ethical Committee approved the research. All methods were carried out in accordance with relevant guidelines and regulations. The patients were instructed to wash and dry their hands, put a pulse oximeter on the index finger, and bend the fingers so that their fingernails were pointing towards them. The recordings were initially completed over a 2 h period and patients remained in rest state. The same subjects were approached two months after their recovery, and the same data collection process was repeated. The recordings fulfilled the inclusion criteria such as no previous history of pulmonary or cardiovascular problem, recordings > 2 h, containing recordings of both during and of recovery were included in the study. The recordings were scanned for artifacts and replaced with mean values using a zero-line interpolation, which is the most stable method compared to other methods of artifact removal [16]. The smallest number of data points in a file was 7421, which were chosen for each recording to perform pattern analysis of OSV signals using Haar wavelet and different scale-based entropy metrics. Boghal and Mani [16] used 1 h recordings of OSV signals to test the integrity of CRCS in young and elderly healthy individuals. Long duration recordings improve the statistical reliability of evaluated metrics. As the subjects had to remain at rest during the data collection, we were able to record data of more than two hours, which is sufficient for performing pattern analysis of OSV signals.

### 3.2. Discrete Wavelet Transform

Consider the following discrete time series containing *N* equally spaced samples of an analog signal:

(1) $$F=[f_{1},\;f_{2},\;f_{3},\;\ldots ,\;f_{N}]$$

Wavelet transform converts this signal to two signals of half its length. The first sub-signal is the running average, or approximation of the time series, and shows the trend of data, whereas the second sub-signal is a running difference that shows fluctuations or the details of the local variations that occur in the data. There have been different types of operations proposed for this transformation, but the key function of all of them is to find one smooth version of the original signal and another version that contains the local details. We can describe these transformations as follows:

(2) $$A=Trend\left(F\right)\;D=Fluctuation\left(F\right)$$

**Figure 1:**
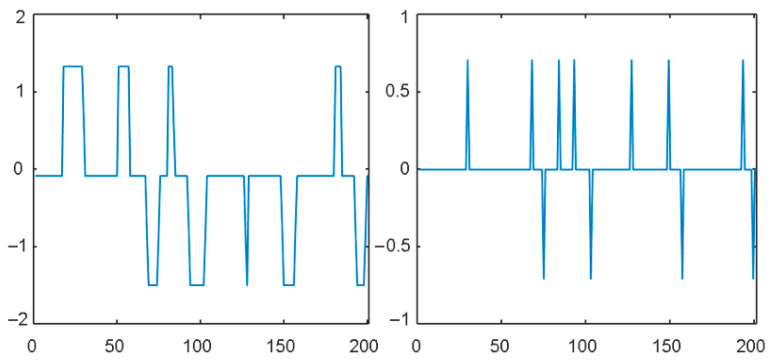
Trend and fluctuation sub-signals of OSV time series.

These sub-signals of a part of a typical OSV time-series signal are shown in Figure 1.

The trend signal can be transformed again using the same procedure to obtain the second stagSe of the trend and fluctuation signals. Depending on the application in hand, this process can be repeated for as many stages as required. We can describe this process of signal decomposition as below:

(3) $$F=\left[A_{1\;}|\;D_{1}\right]=\left[A_{2\;}|\;D_{2}\;|\;D_{1}\right]=\left[A_{3}\;\right|\;D_{3}\;\left|\;D_{2}\;\right|\;D_{1}]=\ldots$$

where the subscripts added to the trend and fluctuation signals indicate their stage. Note that the sub-signals shown at any stage can be combined to retrieve the original signal *F*.

### 3.3. Proposed Method

The Haar wavelet is the simplest of all wavelets, and it simply takes the sum and difference of adjacent samples to calculate the trend and the fluctuation sub-signals. The elements of the trend and fluctuation sub-signals of the input signal *F* can be represented respectively as follows:

(4) $$A\left(m\right)=\left(F\left(2m-1\right)+F\left(2m\right)\right)/\sqrt{2},\;D\left(m\right)=\left(F\left(2m-1\right)-F\left(2m\right)\right)/\sqrt{2},\;m=1,2,3,\ldots ,N/2$$

Note that the Haar transformation divides the output by the square root of 2 to preserve the signal’s energy; however, we can carry out our analysis without this division for simplicity, as we are interested only in the analysis of the structure of signals. This can improve computational efficiency.

The integrity of the cardio–respiratory control system has been hypothesized that it can be established by analyzing the trend and fluctuation signals obtained through wavelet transformation of the OSV time series. Several statistical measures can test the structural complexity of these signals, including amplitude, mean value, number (frequency) of peaks over time, etc. We experimented with several of them and found that variation in the trend signal represents well the complexity of the signal.

We subtract the mean value of the trend sub-signal from its original samples computed in (4) to eliminate the effect of any bias induced by measurement or a subsequent operation.

The times of the zero-mean signal change signs are counted from negative to positive or vice versa. This measure is found to reliably distinguish between the signals coming from the patients of COVID-19 disease and those who have recovered. This step finds the zero crossings of the trend signal. For this, first, we calculate the sign of each element in the trend sub-signal *A*:

(5) $$\;S\left(m\right)=\left\{\begin{array}{cc} 1, & A(m)*A(m-1)<0\\ 0, & otherwise,\;m=1,2,3,\ldots ,\;N/2 \end{array}\right.\;C=\sum_{m=1}^{N/2-1}S(m)$$

The value *C* gives a measure of the structural complexity of signal *A*.

### 3.4. Complete Proposed Algorithm

**Figure 2:**
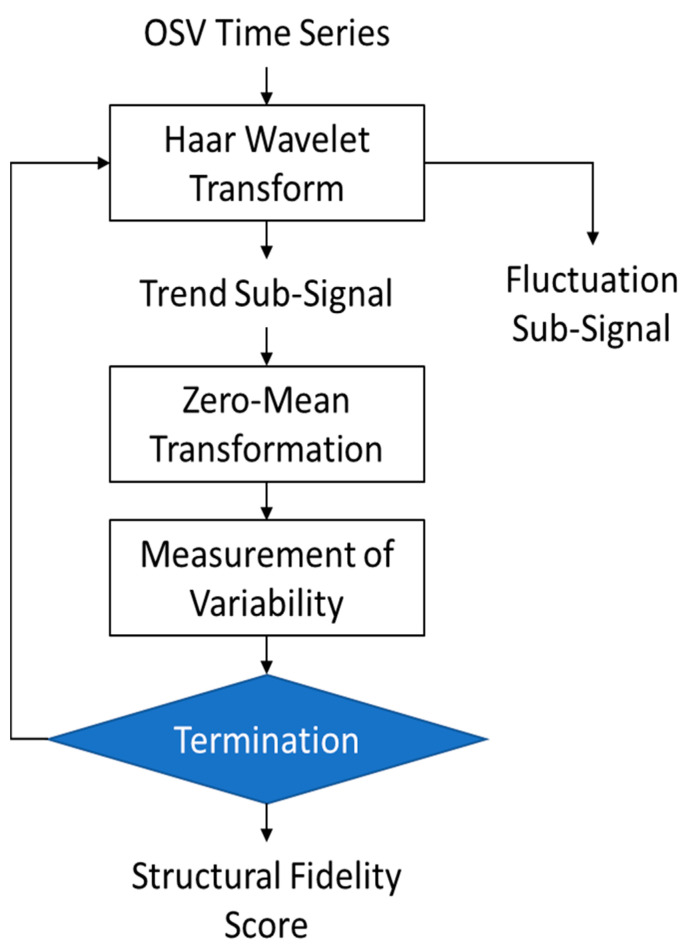
The flow chart of the proposed algorithm.

The complete proposed algorithm comprising the operations described above is shown in Figure 2 and can be summarized as follows:

1. Apply the Haar Transform to the OSV time series and convert it into two sub-signals, trend, and fluctuations. Apply this function iteratively five times to obtain the sub-signals up to stage 5.
  (6) $$\begin{array}{ll} OSV & =\left[A_{1}\;\right|\;D_{1}]\\ & =\left[A_{2}\;\right|\;D_{2}\;|\;D_{1}]\\ & =\left[A_{3}\;|\;D_{3}\right|\;D_{2}\left|\;D_{1}\right]\\ & =\left[A_{4}\;\left|\;D_{4}\;\right|D_{3}\right|\;D_{2}\left|\;D_{1}\right]\\ & =\left[A_{5}\;|\;D_{5}\left|\;D_{4}\;\right|D_{3}\right|\;D_{2}\left|\;D_{1}\right] \end{array}$$
2. Discard the fluctuation sub-signals and perform analysis on the trend signals only, i.e., ${[A}_{1},\;A_{2},\;A_{3},\;A_{4},\;A_{5}]$ computed in the previous step.
3. The mean value of each trend sub-signal is subtracted to eliminate biases and normalize all five sub-signals to zero-mean form, as given below:
  (7) $$A_{z1}=A_{1}-mean\left(A_{1}\right),\;A_{z2}=A_{2}-mean\left(A_{2}\right),\;\;A_{z3}{=A}_{3}-mean\left(A_{3}\right),\;\;A_{z4}{=A}_{4}-mean\left(A_{4}\right),\;A_{z5}{=A}_{5}-mean\left(A_{5}\right)$$
4. The next step is the measurement of variability in each of the zero-mean trend sub-signals using the method of counting zero-crossings mentioned earlier in Equation (5).
5. Step 4 yields five values of the variability measure C computed using Equation (5). Their mean value is taken as the overall score of the structural fidelity of the signal.

## 4. Results and Discussions

Various complexity analysis techniques have been utilized in the past to evaluate the structural complexity of time series data. One widely employed approach for assessing the impact of cardio–respiratory system malfunction is the multi-scale entropy (MSE) analysis of oxygen saturation variability (OSV) signals [14]. In this section, we present the results of the proposed Haar wavelet-based technique and compare them with different versions of scale-based entropy measures, namely MSE [14], moving permutation entropy (MPE) [22], moving fuzzy entropy (MFE) [23], and moving amplitude-aware permutation entropy (MAAPE) [21] at temporal scales ranging from 1 to 20.

To begin the multi-scale entropy analysis, the first step involves constructing a coarse-grained signal by applying a moving average to non-overlapping samples of the OSV signal, with the scale parameter determining the number of samples used in the averaging process. Subsequently, the coarse-grained signal is divided into overlapping vectors of samples, where the vector size is determined by a second parameter known as the order. Once the coarse-grained time series is obtained, we compute the complexity of the OSV signals acquired from COVID-19 subjects during the infection and after recovery using entropy estimation techniques such as sample entropy [11], permutation entropy [12], fuzzy entropy [13], and amplitude-aware permutation entropy [29].

**Figure 3:**
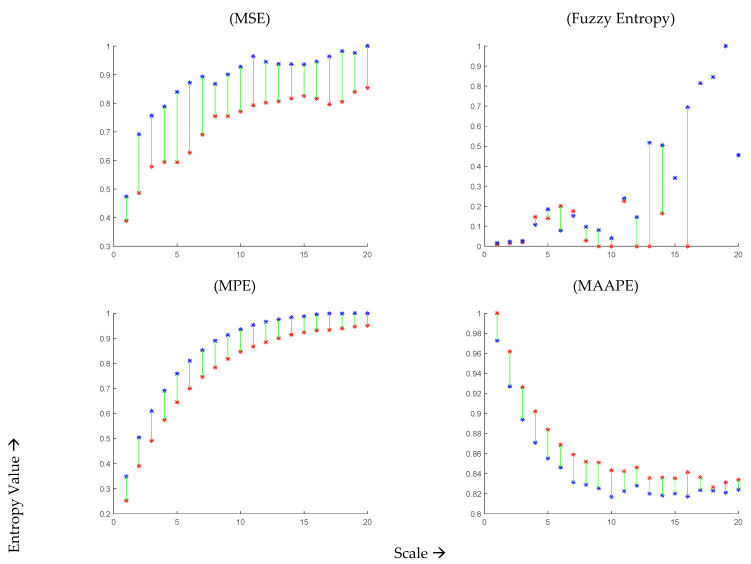
Mean values MSE, MFE, MPE, and MAAPE of 44 COVID-19 subjects at time scale 1 to 20 during infection (red curve) and after recovery (blue curve).

In Figure 3, we present the mean values of MSE (top left panel) for the 44 COVID-19 patients during infection and after recovery at different scales within the range of 1 to 20. It can be observed that the mean entropy value after recovery is consistently higher than that during illness across all scales. The maximum separation between the subjects during and after recovery was observed at time scale 8. However, at very high values, the moving average tends to reduce the structural details, leading to a decrease in the observed differences. The top right panel of Figure 3 displays the results obtained using MFE at time scales 1 to 20. It is worth noting that the fuzzy entropy values become undefined at scales greater than 10, and they exhibit abrupt changes with varying scales.

Moving on to the MPE results shown in the bottom left panel of Figure 3, they aim to differentiate OSV signals during the infection and after two months of recovery from COVID-19 at time scales 1 to 20. It can be observed that the MPE values are consistently smaller during the infection compared to those after two months of recovery. The maximum separation between the subjects during and after recovery was observed at time scale 8. Similarly, the bottom right panel of Figure 3 presents the results obtained using MAAPE, revealing a decrease in complexity during COVID-19 infection and an increase in complexity after recovery. The maximum difference between the subjects during and after COVID-19 infection was observed at time scale 10. Interestingly, unlike other scale-based entropy metrics (MSE, MFE, and MPE), the MAAPE values drop for both infected and recovering classes at higher scales. This behavior can be attributed to the fact that MAAPE considers amplitude information, unlike permutation entropy which focuses on the order of patterns for quantifying signal complexity. In summary, the estimates obtained from all four scale-based entropy measures indicate a loss of complexity during COVID-19 infection and an increase after two months of recovery, which aligns with other studies conducted on various physiological signals [14,21,22], indicating that the loss of complexity is a general characteristic of the disease.

**Figure 4:**
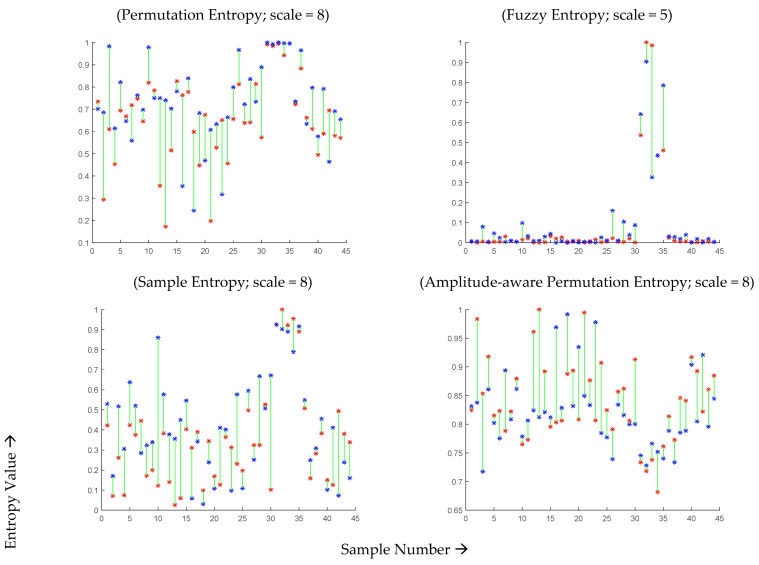
MSE, MFE, MPE, and MAAPE estimates for 44 COVID-19-affected subjects during infection (red points) and two months after recovery (blue points) at optimal time scales.

**Figure 5:**
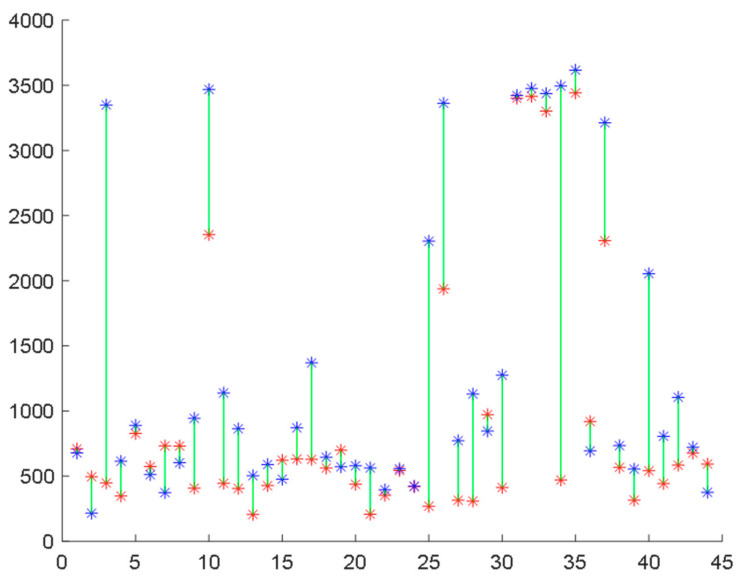
Structural fidelity scores assigned by the proposed method to 44 COVID-19-affected subjects during infection (red points) and after recovery (blue points).

For a more detailed analysis, Figure 4 presents the individual entropy values of the 44 subjects during infection and after recovery. The optimal scale value was selected for each entropy measure based on the analysis of the average scores presented in Figure 4, which exhibited significant differences. For MSE, MFE, MPE, and MAAPE, the entropy estimates showed an increase in 28, 30, 33, and 31 subjects, respectively. Notably, the permutation entropy values of 33 subjects (75% of the total) improved after recovery, while the remaining 11 subjects did not follow this pattern. Although 11 out of 44 may seem like a significant number, it can be argued that these results are promising given the potential data capture errors and the inability to repeat the experiment or further investigate the cases of the minority subjects that did not conform to the observed pattern. Similar trends were observed using other entropy measures, as depicted in Figure 5.

**Figure 6:**
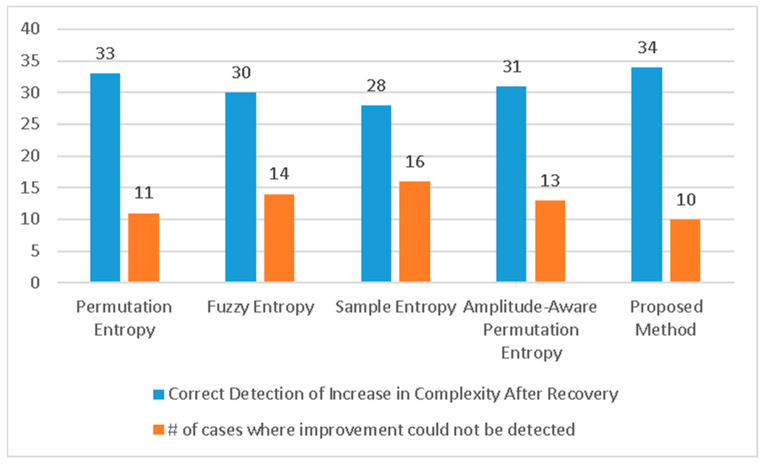
Number of correct and incorrect detections of increase in complexity of signals after recovery. The proposed method has the best performance compared to the existing state-of-the-art algorithms.

Furthermore, we repeated the experiment using the proposed Haar wavelet-based technique and plotted the individual scores assigned by this method to the subjects during infection and after recovery. In this case, 34 subjects (77.3% of all subjects) showed improvement in their scores during COVID-19, while 10 subjects did not exhibit this progress, as illustrated in Figure 5. These results surpass the best outcomes achieved by any form of multi-scale entropy analysis. A comparison of the results is presented in Figure 6.

It is important to note that multi-scale entropies necessitate the specification of several parameters, including scale, order of permutation, delay, similarity threshold, etc. In contrast, the proposed Haar wavelet-based technique does not require any user-defined parameters. This aspect contributes to the simplicity and ease of use of the proposed method, providing additional advantages over the existing multi-scale entropy-based methods.

**Figure 7:**
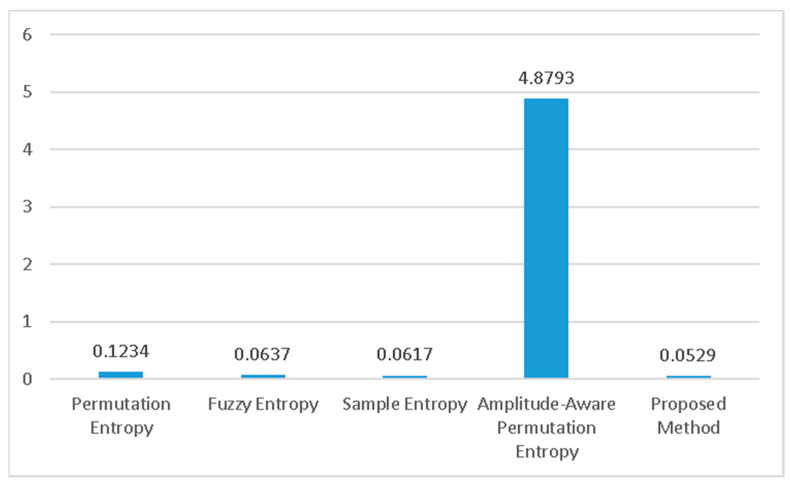
The execution time of different methods. The proposed method is the fastest.

Finally, Figure 7 showcases a comparison of the execution speeds of the various methods being evaluated. The proposed technique proves to be the fastest among all, with an average execution time of only 52.9 milliseconds for one subject’s data. On the other hand, amplitude-aware permutation entropy is the slowest among the compared methods.

A few studies [34,35,36] quantitatively describe oxygen saturation dynamics and patterns in COVID-19 patients using time domain and nonlinear techniques such as approximate entropy and Lempel–Ziv complexity measures. These techniques provided value insights for assessing prevalence of obstructive sleep apnea–hypopnea syndrome, breathing pattern disorder, and assessment of disease severity and response to respiratory support of hospitalized COVID-19 patients. The traditional entropy measures such as approximate entropy and Lempel–Ziv complexity are single scale. Like other physiological signals, OSV signals are the output of numerous interacting sub-components of the cardio–respiratory control systems (CRCS) operating on multiple time scales. Thus, single-scale, traditional entropy measures are unable to accurately yield dynamical information about interacting components of complex biological systems operating on multiple time scales. The scale-based entropy measures such as MSE [14], MPE [22], MFE [23], and MAAPE [21] can have the potential to quantify the complexity of CRCS at multiple time scales using OSV signals. The multi-scaling procedure adds to the computational burden and slows the process of analyzing the complexity of OSV signals [20]. The main contribution of this study was to propose innovative techniques for analyzing the dynamical complexity of OSV signals. The proposed techniques using Haar wavelets not only improved the performance for distinguishing OSV signals during and after recovery, but also was time efficient.

The study also highlighted that complexity of OSV signals of COVID-19 patients decreased during COVID-19 compared to those after two months of recovery. The reduced complexity during COVID-19 reveals the lesser adaptive capability of CRCS to external stresses. After recovery, the adaptive capability of the CRCS started to increase revealing an increase in its dynamical complexity. The proposed technique can have applications in evaluating dynamical models of CRCS and clinical monitoring during disease and after recovery.

One of the major limitations of the study is that the cohort size is modest. In the pilot study data were used for analysis of the dynamical characteristics of OSV signals during COVID-19 and after two months of recovery. However, further studies with a large data set can be useful for accurate clinical decision-making.

## 5. Conclusions

Past research has established that the complexity of the human body’s cardiac–respiratory signals decreases if the related body organs are not in their best health. Since these signals can be measured quickly with simple wearable devices available on the market, such analysis can help take preventive measures and monitor recovery. In this paper, we presented a simple measure that can determine the structural fidelity of the signals using wavelet transform. In addition, a dataset of oxygen saturation levels of 44 COVID-19 patients comprising more than two hours of recording of each subject was collected when they were infected and being treated and then again after two months of their recovery.

The proposed technique was tested on this dataset to validate its accuracy and time efficiency. It was found that 34 out of 44 subjects improved their score regarding the proposed measure. Improvement was not observed for the remaining ten subjects, or their scores were found to have dropped after recovery. Further investigation could help in determining the cause. The subjects might still not be fully recovered, or some noise might have affected the data collection process. Nevertheless, 34 out of 44 is a significant number which makes the proposed technique a candidate to be seriously considered for further investigations on larger data and as a tool for observing the recovery of patients.

## Data Availability

Not applicable.
